# Alzheimer disease neuropathology: understanding autonomic dysfunction

**DOI:** 10.1590/S1980-57642009DN20300004

**Published:** 2008

**Authors:** Eliasz Engelhardt, Jerson Laks

**Affiliations:** 1 MD, PhD, Coordinator of the Cognitive and Behavioral Unit – INDC-UFRJ.; 2 MD, PhD, Coordinator of the Alzheimer’s Disease Unit – CDA/IPUB-UFRJ.

**Keywords:** Alzheimer, neurodegeneration, autonomic, autonomic dysfunction, dysautonomy

## Abstract

Alzheimer’s disease is a widely studied disorder with research focusing on cognitive and functional impairments, behavioral and psychological symptoms, and on abnormal motor manifestations. Despite the importance of autonomic dysfunctions they have received less attention in systematic studies. The underlying neurodegenerative process of AD, mainly affecting cortical areas, has been studied for more than one century. However, autonomic-related structures have not been studied neuropathologically with the same intensity. The autonomic nervous system governs normal visceral functions, and its activity is expressed in relation to homeostatic needs of the organism’s current physical and mental activities. The disease process leads to autonomic dysfunction or dysautonomy possibly linked to increased rates of morbidity and mortality.

**Objective:**

The aim of this review was to analyze the cortical, subcortical, and more caudal autonomic-related regions, and the specific neurodegenerative process in Alzheimer’s disease that affects these structures.

**Methods:**

A search for papers addressing autonomic related-structures affected by Alzheimer’s degeneration, and under normal condition was performed through MedLine, PsycInfo and Lilacs, on the bibliographical references of papers of interest, together with a manual search for classic studies in older journals and books, spanning over a century of publications.

**Results:**

The main central autonomic-related structures are described, including cortical areas, subcortical structures (amygdala, thalamus, hypothalamus, brainstem, cerebellum) and spinal cord. They constitute autonomic neural networks that underpin vital functions. These same structures, affected by specific Alzheimer’s disease neurodegeneration, were also described in detail. The autonomic-related structures present variable neurodegenerative changes that develop progressively according to the degenerative stages described by Braak and Braak.

**Conclusion:**

The neural networks constituted by the central autonomic-related structures, when damaged by progressive neurodegeneration, represent the neuropathological substrate of autonomic dysfunction. The presence of this dysfunction and its possible relationship with higher rates of morbidity, and perhaps of mortality, in affected subjects must be kept in mind when managing Alzheimer’s patients.

## Abstract (pt)

A doença da Alzheimer é uma doença amplamente estudada com foco nos comprometimentos cognitivo e funcional, sintomas de comportamento e psicológicos e manifestações motoras anormais. As disfunções autônomas, apesar de sua importância, foram menos consideradas por estudos sistemáticos. O processo neurodegenerativo subjacente dessa doença, principalmente nas áreas corticais, vem sendo estudado há mais de um século. Entretanto, estruturas autônomas não foram estudadas do ponto de vista neuropatológico com o mesmo interesse. O sistema nervoso autônomo encontra-se relacionado a funções viscerais normais e sua atividade é expressa em relação a necessidades homeostáticas das atividades correntes físicas e mentais do organismo. O processo da doença leva a disfunção autônoma ou disautonomia, possivelmente relacionada com taxas maiores de morbidade e mortalidade.

**Objetivo:**

O foco dessa revisão é analisar as estruturas autônomas corticais, subcorticais e mais caudais, assim como o processo neurodegenerativo específico da doença de Alzheimer que acomete essas estruturas.

**Métodos:**

Foi realizada busca de artigos sobre estruturas autônomas atingidas pela degeneração de Alzheimer e em condições de normalidade, através do MedLine, PsycInfo e Lilacs, assim como nas referências bibliográficas dos artigos de interesse encontrados, busca manual de estudos clássico em periódicos e livros mais antigos, abrangendo mais de um século de publicações.

**Resultados:**

As principais estruturas autônomas centrais são analisadas do ponto de vista funcional, incluindo áreas corticais, estruturas subcorticais (amígdala, tálamo, hipotálamo, tronco cerebral) e medula. Estas constituem as redes neurais autônomas subjacentes às funções vitais. As mesmas estruturas, atingidas pela neurodegeneração específica da doença de Alzheimer foram também descritas de modo detalhado. As estruturas autônomas apresentam alterações neurodegenerativas de grau variável que se desenvolvem de modo progressivo de acordo com os estágios degenerativos descritos por Braak e Braak.

**Conclusão:**

As redes neurais constituídas pelas estruturas autônomas centrais, quando lesadas pela neurodegeneração progressive, representam o substrato neuropatológico da disfunção autônoma. A presença dessa disfunção e sua possível relação com taxas mais elevadas de morbidade e talvez de mortalidade entre os indivíduos comprometidos deve ser considerada quando se trata de pacientes com doença de Alzheimer.

The autonomic nervous system (ANS) is responsible for the coordinated control of several visceral systems, such as the cardiovascular, respiratory, and digestive systems. Under normal conditions, this function is adjusted aiming at homeostasis as a response to physical efforts. It is also adjusted in relation to changing mental activities. In this sense it can be said that in a normal organism, somatic and mental (emotional, cognitive) activities are always accompanied by adjustments in ANS control. The regulation of control enables these complex activities to meet their basic needs. The two extreme paradigms of mobilization of the ANS are represented by the “quiet-rest” and “fight-and-flight” states. A broad spectrum of intermediary possibilities which occur in daily life lay between these two states, both during wakefulness and during the several stages of sleep. The mobilization of the ANS is always graded according to the current needs of the situation.^1-10^

Damage to the ANS that impairs function beyond compensatory limits may yield dysfunctional states in several organs and systems, representing a picture of “autonomic dysfunction” or “dysautonomy”. These disorders may manifest themselves as autonomic hyperactivity (e.g. hypertension, arrhythmias, hyperhidrosis), or as autonomic failure (e.g., orthostatic hypotension, gastrointestinal tract hypomotility, incontinence). Some of these manifestations may be asymptomatic and detectable only on clinical examination or autonomic testing. Others may be life threatening, such as ventricular arrhythmias, or can cause severe impairment in daily activities, such as orthostatic hypotension. In general, autonomic hyperactivity tends to occur in the context of acute neurologic disease (e.g. cerebrovascular ictus), whereas neurodegenerative disorders (e.g. the well-studied Parkinson’s disease and Dementia with Lewy Bodies, and the less studied Alzheimer’s disease) are commonly associated with autonomic failure. Autonomic responses to cognitive challenge and emotionally significant stimuli may also be impaired, indicating a defective linkage between mental status and autonomic responses, which is important in the pathogenesis of autonomic dysfunction. It is possible that autonomic dysfunction in Alzheimer’s disease is related to the higher rates of morbidity (due to cardiovascular, respiratory, gastrointestinal, bladder disorders) and mortality observed among the affected patients.^5,11-14^

The objective of the present review was twofold. First, the general constituents and integrative aspects of the central autonomic nervous system were discussed, followed by a description of the main autonomic-related structures and their interconnections under normal conditions. Second, a systematic description of the same autonomic-related structures affected by Alzheimer’s neurodegenerative pathology was provided. Lastly, some considerations were presented to understand the damaged structures from a functional viewpoint in the context of derranged autonomic neural networks, as well as the clinical repercussions in the light of the autonomic dysfunction.

## Methods

A broad search spanning over one century was performed for papers investigating autonomic-related structures affected by Alzheimer’s neuropathology, and the same structures studied from normal anatomical and functional viewpoints. Sources included MedLine, PsycInfo and Lilacs, as well as the bibliographical references of the papers of interest. In a further effort to locate relevant information, a manual search for classical studies in older journals and books was also performed. This search included papers written in several languages (English, German, French, Spanish and Portuguese).

### The autonomic nervous system

The ANS comprises central and peripheral structures. The central structures are constituted by three components that integrate complex autonomic functional patterns, and associate cognitive and behavioral manifestations with autonomic expression (i) nuclei that contain preganglionic efferent neurons localized in the brainstem and spinal cord, whose axons innervate autonomic ganglia, sympathetic and parasympathetic, and modulate the enteric nervous system, (ii) autonomic nuclei, with graded complexity, related to control and to pattern generation, localized in the hypothalamus and brainstem, besides the amygdala, thalamus, striatum, and cerebellum, and (iii) selected cortical areas.

These central autonomic regions are widely interconnected and constitute a complex network, with tonic, reflex and adaptive control over autonomic functions. Additionally, it regulates endocrine, behavioral, and other responses. The central ANS maintains continuity with the peripheral autonomic structures through which a delicate control is exerted on autonomic effectors of the viscera. Activity within the central ANS is state-dependent and affected by internal and external influences.^15-16^

The highly integrated patterns of autonomic functions are mostly generated in core structures including the hypothalamus (proposed as the main integrator by pioneer researchers) and the brainstem. The autonomic activities, as well as the endocrine and behavioral components of a given response, are exerted in a temporal and spatial sequence. A combination of responses of a more limited pattern with higher levels of organization results in the necessary adjustments of autonomic control. The hypothalamus may determine the overall characteristics of the response (and how it will fit with ongoing needs), whereas subsidiary pattern generators may each produce a series of response patterns, with graded complexities. The more complex generators exert control on more elementary autonomic (and endocrine, and motor) actions. When engaged in different combinations, these organized generators can produce the entire range of highly differentiated responses necessary to maintain homeostasis and other vital functions. The different pattern generators at varied levels of the neuraxis are organized in a hierarchical manner, so that they allow for individual response patterns to become part of larger responses, where the resultant action is arises from multiple level integration of autonomic pattern generators.^2,15^

### Central autonomic structures and their interrelationships

The central autonomic regions include cortical areas, subcortical regions, brainstem, and spinal cord structures. Evidence obtained from neuroanatomical, lesional, electrophysiological, and functional studies indicate the role of several cortical areas in central autonomic modulation, including medial posterior frontal and posterior orbitofrontal areas, anterior cingulate area and insular cortex. These areas are the only known sources of projections from the cortex directly to subcortical autonomic centers, such as the hypothalamus and brainstem, and in addition, are reciprocally connected with limbic-paralimbic and heteromodal association areas.^5,16-23^

The subcortical autonomic centers are integrative regions where pattern generators of varied complexity are located, mainly in the hypothalamus and brainstem.^15^ The hypothalamus is a key regulatory center for autonomic and endocrine-metabolic control, and links the highest with the lowest levels of the neuroaxis.^24-26^ The brainstem is one of the most important regions regarding autonomic vital functions as it incorporates the respiratory, cardiovascular, and gastrointestinal control centers, represented by numerous nuclei (higher order processing autonomic nuclei [control and pattern generation], including periaqueductal gray (PAG), reticular formation nuclei (RF), nucleus tractus solitarius (NTS), parabrachial (PB) nuclear complex and dorsal motor vagal nucleus (DVN). The parasympathetic preganglionic neurons, including the pupillary (Edinger-Wetphal), salivatory (superior and inferior), and the DNV are also located here.^27-30^ Other subcortical structures of variable complexity, also play a role in autonomic integration, including the amygdala^26,31-32^ limbic and midline thalamic nuclei,^33-36^ accumbens,^37^ and the cerebellum, considering the cerebellar cortical-deep nuclei modules of the median region as a unit.^38-42^ All subcortical autonomic-related structures are widely interconnected, and relay cephalad projections to the cerebral cortex, and caudad projections to the spinal cord. In the spinal cord the preganglionic neurons are localized at the thoracolumbar [sympathetic] and sacral [parasympathetic] levels. The preganglionic neurons of the brainstem and spinal cord innervate the parasympathetic and sympathetic autonomic ganglia and the enteric nervous system which are directly related to cephalic, thoracic, abdominal and pelvic autonomic effectors.^24,43-46^ These regions participate in the constitution of complex neural networks linking high-level cognitive and affective sites with lower integrating structures to influence autonomic, emotional and behavioral responses.^15^

### The neurodegeneration of central autonomic-related structures in Alzheimer’s disease

The main neuropathological markers of AD have been known for more than a century.^47-49^ They include senile plaques (SPs) and neurofibrillary tangles (NFTs), in varied stages of development. These changes follow a characteristic sequence, where, at the cortical level, the hippocampal formation is the earliest affected structure, followed progressively by other allocortical and finally, neocortical areas.^50,51^ Besides the well studied cortical areas there are the less studied subcortical structures that may be equally affected by the neurodegeneration, but for which less information is available.

The basic markers, namely neurofibrillary degeneration (NFTs, including neuropil threads, and components of neuritic plaques) and SPs (seen in varied stages of formation, presenting dystrophic neurites in mature SPs – the neuritic plaques) characteristic of this neurodegenerative process, are not seen homogeneously across the several neural levels. There can be a predominance of one of the other, according to the examined region. The distribution pattern of SPs is different to NFTs. SPs commonly present a patchy distribution and a varied density, even considering the architectonic limits of the several areas. The inconsistent presence, varied density and pattern of distribution of SPs preclude the use of this marker for reliable neuropathological staging of the disease. On the other hand, NFTs present a well-defined sequential pattern, permitting differentiation of stages, and show better correlation with severity of the disease.^52-58^ The high number of NFTs, not the volume of amyloid deposits, corresponds to the reduction of the number of neurons in all studied areas. This progressive neuronal loss, as well as interconnections, is accompanied by functional impairment expressed as clinical symptoms of the disease.^54,59^ These long-known markers, revealed by the classic staining techniques (aniline, silver) still in use today, are visible on optical microscopy. Recently, numerous techniques have been developed which are now used in addition to the classical techniques to verify neuropathological aspects linked to the neurodegenerative process. The newer staining techniques, including immunological variants, allow earlier and more detailed visualization of the pathological material (thioflavine S and anti-beta/A4 [for amyloid], mab tau-1, mab-Alz50, AT8 and anti-PHF serum 60e, mab 3-39 to PHF [for tau and abnormally phosphorylated tau], mab 3-39 to PHF, which recognizes the carboxy terminal domain of ubiquitin). The immunocytochemical methods (such as AT8) permit assessment of neuronal changes antedating the formation of NFTs, and follow, in the same way, the sequence and stages previously established with the classic techniques.^51,52,60,61^

The information that follows takes into account the presence of neurodegeneration of the affected structures irrespective of the techniques employed to show the pathological changes, unless they are necessary for a better understanding. Morphometric is included whenever available and the specific pathological changes in the described structures will be related to the neurofibrillary degenerative Braak and Braak’s stages, whenever available. This staging is accepted and used by most authors as the best pathological staging system and a time-line for the progression of the disease.

### Cerebral cortex

The cortical areas are known to be affected by the neurodegeneration in a progressive and sequential manner, classified as I-II (transentorhinal), III-IV (limbic) and V-VI (isocortical) Braak and Braak stages. The changes first emerge in entorhinal areas (stage I-II), and progress to other limbic and paralimbic structures (the more severely affected), followed by heteromodal associative structures. The autonomic-related areas (prefrontal medial and orbitary), the anterior part of the cingulate gyrus, and the insular cortex become progressively involved from stage III-IV, reaching maximum severity at stage V-VI. The severity is higher than in any associative areas of the frontal, parietal and occipital lobes, and comparable to the involvement of the temporal cortex.^5,14,20,60,62^

### Subcortical structures

The autonomic-related subcortical structures include the amygdala, thalamus, basal ganglia, hypothalamus, and cerebellum.

#### Amygdala

Strong neurodegenerative changes are seen early in the disease (stage II-III), attaining the highest degree of neurofibrillary degeneration in the more advanced stages. The degeneration was seen in the cortical, mediobasal, lateral, basal accessory, lateral basal and central nuclei, but there is no consensus among authors about the intensity of lesions in the several nuclei, possibly due to the restricted number of cases in each study.^63-65^ Morphometric data also contribute toward evaluating the degree of the degenerative lesions. Such studies show that the amygdala and its subnuclei undergo severe volumetric atrophy. Total numbers of neurons were reduced significantly where medium and large neurons were predominantly affected. There is a neuron loss of about 50% in each amygdala. The subdivisions showed a differential neuron loss ranging from 35% in nucleus lateralis to 70% in the basalis.^66,67^

#### Thalamus

Severe changes were confined to some of the limbic nuclei. The anterior (anterodorsal), dorsomedial, and laterodorsal nuclei showed changes early in the disease process (stage I-II), and reached heavy NFT burden from stage V onwards. The anteroventral nucleus presents changes at stage IV and reaches maximum burden at stage VI. Among the midline nuclei, the paraventricular and reuniens began NFTs load at stage IV and peaked at stage VI. Other nuclei were affected in later stages.^53,68-72^

#### Basal ganglia

Neurofibrillary degeneration was present throughout the striatum, but displayed significantly higher densities in the ventral part (nucleus accumbens and olfactory tubercle). The changes began at stage III-IV and became more severe at VI. The dorsal striatum was affected later, at stage V-VI. No neurodegeneration was found in the globus pallidus. These findings suggest that the ‘limbic’ striatum is particularly vulnerable to AD pathology.^73-76^

#### Hypothalamus

Several of its regions and nuclei are severely affected by the neurodegenerative process. The various nuclei are not involved simultaneously and show different staining patterns. The intensity also varied among the reports, probably due to different severity stages examined and different staining methods. The lateral hypothalamus (tuberomammilar, lateral tuberal, posterior) was affected from stage IV onwards and affected maximally at stage VI; supraoptic (supraoptic, paraventricular), followed in severity by the mediobasal hypothalamus (dorsomedial, ventromedial, tuberomammilar, lateral tuberal, tuberoinfundibular nuclei, tuberal grey, periventricular), and the anterior hypothalamus (sexually dimorphic and suprachiasmatic nuclei, periventricular area, arcuate, median eminence) was affected less intensely and only in later stages.^10,61,70,77-81^

#### Cerebellum

The cerebellum had long been a relatively neglected area of the AD brain, believed to be unaffected by specific neuropathology. A number of studies, although controversial, have revealed that pathological changes are present. Neurodegenerative changes have been observed, such as amyloid deposits (diffuse plaques, compact plaques), but NFTs were absent. The majority of plaques occur in the molecular layer, extending to the Purkinje cell layer and seldom into the granular layer.^82-85^ Morphometric data, in comparison to controls, provides some clarification. There was, in severe cases, a decrease in the volume of the molecular (24%) and granular (22%) layers. A reduction in the total number of Purkinje cells (32%) was seen to correlate with atrophy of the molecular layer. There was a similar reduction in the total number of granule cells (30%), which correlated with atrophy of the molecular and granular layers. Purkinje cell density, measured in the vermis and cerebellar hemispheres, showed the mean number of these cells to be significantly decreased in the vermis. Atrophy in the vermis was also more severe. The correlation between the temporal duration and both cortical neuronal and volumetric losses of the molecular and granular cortical layers indicate that these cerebellar atrophic changes most likely represent the disease process involving mainly the vermis.^86,87^

#### Brainstem and spinal cord

The neurodegenerative process affects the brainstem in a heterogeneous manner, displaying a decreasing rostrocaudal gradient while affecting more superior and dorsal regions and reaching the lowest degree in the most caudal segments, where it meets the spinal cord. The specific pathology is well expressed in these structures. Although there were no data on stage-related changes, the duration of dementia of the studied cases ranged between 2 and 17 years, possibly including all stages of the disease.

The majority of cranial nerve nuclei are generally spared. Only those that belong to the cranial autonomic parasympathetic outflow are clearly affected, including the pupillary nucleus (Edinger-Westphal) and the DNV, that give rise to parasympathetic preganglionic fibers, display a marked neuronal loss. Additionally, the NTS, an important afferent centre (for the VII, IX, X nerves) and relay station for several autonomic reflexes (cardiovascular, respiratory, gastrointestinal), besides the nucleus ambiguous that innervates muscles of branchial origin (IX, X, XI nerves), are also affected.^57,88,89^

The aminergic mesencephalic (dopaminergic of the substantia nigra compacta and ventral tegmental area), pontomesencephalic (serotonergic, cholinergic, noradrenergic nuclei), and medullary (adrenergic) are clearly affected.^57,77,90,91^

Several nuclei of the RF are affected in the mesencephalon, pons (tegmentopontine, oral and caudal), and medulla (medial and lateral). They are related to several mechanisms, including cardiovascular and respiratory control, swallowing, defecation and urination.^55,57,88,92-95^

The more complex autonomic centers such as the PAG, pontine PB complex, and intermediate reticular zone (IRZ) of the medulla, show neurodegenerative lesions of variable intensity. The nuclei of the PB complex together with the IRZ are pivotal relay stations within central autonomic regulatory feedback systems. The nuclei of the PB region and the IRZ display specific pathology at stage I-II that corresponds to the preclinical phase of AD. In stage III-IV (mild AD) these nuclei are already severely affected. In stage V-VI (moderate and severe AD), PB nuclei are filled with abnormal intraneuronal material, and the IRZ shows severe damage. The state of the AD-related neurodegeneration of the nuclei of the PB and the IRZ conforms to the cortical neurofibrillary I-VI staging.^88,96^

The spinal cord in AD was seldom studied. There are a few reports, mainly from the older literature, generally without mentioning specific pathology (in AD cases) or with scant or inconsistent findings occurring in most advanced stages of the disease. A very small number of tangles were found infrequently in the central region and intermediolateral column (origin of sympathetic preganglionic fibers), and occasionally in the dorsal and ventral gray in a small proportion of cases (‘senile dementia’). The presence of fatty degeneration and pigmentary accumulation (lipofuscine) in the smaller and larger neurons of the anterior horn, with non-specific fibrillary changes was also described. Thus, neurodegenerative changes were considered to be absent in the spinal cord.^47-49^ More recent papers have focused on the spinal cord. One of these reports that the spinal cord exhibits little or no pathological changes in AD. Another study, which investigated tau-related pathology, detected tau immunoreactivity in neurons of the anterior horn (some with NFTs), but less frequently seen in the intermediate zone and posterior horn. Other regions (intermediolateral column and Onuf’s nucleus) showed no tau pathology. These abnormal cytoskeletal changes were more consistently observed in the cervical enlargement, followed by the thoracic cord and to a lesser extent in the lumbar enlargement. Finally, one paper described pathological tau in the spinal cord, in addition to less frequently seen neurofibrillary lesions. These lesions were most frequently found in the substantia intermedia, and also occurred in the lateral and dorsal horns, as well as in the ventral horns (more often in small neurons and less frequently in anterior horn cells).^55,57,97-100^

#### Concluding remarks

Alzheimer’s disease is a multi-faceted disorder best known for its cognitive, behavioral and motor dysfunctions. Despite its importance, autonomic impairment has received far less attention.

The autonomic nervous system, in its normal state governs visceral functions, and its activity is expressed in relation to homeostatic needs of the organism considering its current physical and mental activities. However, if these functions and their compensatory mechanisms begin to fail, autonomic dysfunction or dysautonomy ensues.

The normal anatomy, connections, and function of the central autonomic-related structures have been reasonably well studied. They constitute neural networks of varied complexity and with a hierarchical nature that underpin vital functions. These structures span from the cerebral cortex to the spinal cord, and include several important subcortical structures (amygdala, thalamus, hypothalamus, brainstem, cerebellum).

However, the neurodegenerative pathology of these autonomic-related structures have not been studied with the same intensity as the cerebral cortex, and only isolated studies partially describing this important issue were found. The present review pools practically all studies found on the central autonomic-related structures affected by specific Alzheimer’s disease neurodegeneration together and describes them in a detailed and systematic way. The present description of these less studied neuropathologically affected regions followed the same sequence used for the relatively well-known structures and their networks in normal states so as to facilitate (dys) functional understanding. All encephalic levels, with the spinal cord practically spared, are affected by the neurodegenerative process to varying degrees, from mild to severe. There seems to be a sequential progression that parallels the Braak and Braak stages, but this was not always revealed in view of the limited data available.

The neurodegenerative changes impair function and lead to death of the involved neurons. Consequently, the autonomic mechanisms progressively deteriorate commensurate with the advancing cortical neurofibrillary stages, causing autonomic dysfunction. The affected neural networks, when damaged by the progressive neurodegeneration, fail to maintain their specific vital activities. Thus they represent the neuropathological substrate of autonomic dysfunction.

This dysautonomy may be expressed through clinical manifestations in the autonomic range which may appear from the beginning of the clinical phase of the disease and progress to more severe phases. The knowledge that a dysautonomic state perpetuates throughout the course of the disease is pivotal for its recognition (clinical or subclinical), and for the introduction of possible corrective measures. The appearance of adverse events upon use of therapeutic agents can also be correctly interpreted. The presence of this dysfunction and its possible link to higher morbidity, and perhaps mortality, in affected subjects must be kept in mind when managing Alzheimer patients.

Therefore, in parallel to well established knowledge of the cognitive, behavioral, psychological, and motor manifestations, it seems paramount to dedicate similar attention to the autonomic dysfunction of Alzheimer’s disease.
